# Supplementary material for: Single Cell Transcriptomic and Chromatin Profiles Suggest Layer Vb Is the Only Layer With Shared Excitatory Cell Types in the Medial and Lateral Entorhinal Cortex
Source: Front Neural Circuits. 2022 Jan 26;15:806154. doi: 10.3389/fncir.2021.806154 (PMC8826650; doi:10.3389/fncir.2021.806154)
Supplement: Supplementary file 14 [file Data_Sheet_1.ZIP › Deep_vs_Sup_Motifs/homerResults/motif11.similar.html]

motif11

## Information for motif11

A
C
G
T
T
C
G
A
C
A
G
T
A
G
T
C
T
A
C
G
T
G
C
A
G
C
A
T
G
A
C
T
G
C
T
A
G
A
C
T
  
Reverse Opposite:  

C
T
G
A
C
G
A
T
C
T
G
A
C
G
T
A
A
C
G
T
A
T
G
C
T
C
A
G
G
T
C
A
A
G
C
T
T
G
C
A
  

|  |  |
| --- | --- |
| p-value: | 1e-34 |
| log p-value: | -7.842e+01 |
| Information Content per bp: | 1.439 |
| Number of Target Sequences with motif | 4389.0 |
| Percentage of Target Sequences with motif | 47.20% |
| Number of Background Sequences with motif | 13326.4 |
| Percentage of Background Sequences with motif | 40.91% |
| Average Position of motif in Targets | 354.5 +/- 256.3bp |
| Average Position of motif in Background | 336.9 +/- 266.4bp |
| Strand Bias (log2 ratio + to - strand density) | -0.0 |
| Multiplicity (# of sites on avg that occur together) | 1.37 |
| Motif File: | file (matrix) reverse opposite |

### Similar de novo motifs found

|  |  |  |  |  |  |  |  |
| --- | --- | --- | --- | --- | --- | --- | --- |
| Rank | Match Score | Redundant Motif | P-value | log P-value | % of Targets | % of Background | Motif file |
| 1 | 0.722 | G A C T A G C T A T G C A C T G G T C A G A C T A G T C C A T G | 1e-28 | -64.841613 | 16.13% | 12.21% | motif file (matrix) |
| 2 | 0.634 | T A G C T C A G T G C A G C T A A C G T A T G C T C A G G C T A C G A T C G A T | 1e-23 | -54.762346 | 11.41% | 8.35% | motif file (matrix) |
